# 2152. Activity of Imipenem/Relebactam Against Carbapenem-Resistant Non-*Morganellaceae* Enterobacterales (NME) in the United States – SMART 2019-2021

**DOI:** 10.1093/ofid/ofad500.1775

**Published:** 2023-11-27

**Authors:** Sibylle Lob, Mark G Wise, Karri Bauer, Fakhar Siddiqui, David W Hilbert, John Esterly, C Andrew DeRyke, Katherine Young, Mary Motyl, Daniel F Sahm

**Affiliations:** Merck & Co., Inc., Schaumburg, Illinois; IHMA, Schaumburg, Illinois; Merck & Co., Inc., Schaumburg, Illinois; Merck & Co., Inc., Schaumburg, Illinois; Merck Research Laboratories, Rahway, New Jersey; Merck & Co., Inc., Schaumburg, Illinois; Merck & Co., Inc., Schaumburg, Illinois; Merck, Rahway, New Jersey; Merck, Rahway, New Jersey; IHMA, Schaumburg, Illinois

## Abstract

**Background:**

Antimicrobial resistance has been increasing among gram-negative pathogens. Carbapenem resistance is especially concerning, as carbapenems are a mainstay of therapy for resistant infections. KPC production is the most common cause of carbapenem resistance in the US, but it can also be mediated by mechanisms such as porin loss or efflux. Imipenem/relebactam (IMI/REL) combines imipenem/cilastatin with relebactam, an inhibitor of class A and C β-lactamases. IMI/REL’s spectrum of activity makes it a therapeutic option for infections with carbapenemase producers and isolates resistant due to other mechanisms. We evaluated the activity of IMI/REL and comparators against carbapenem-resistant (CR) non-*Morganellaceae* Enterobacterales (NME) isolates collected from patients in the US as part of the SMART surveillance program.

**Methods:**

In 2019-2021, 24 US clinical labs collected up to 250 consecutive, aerobic or facultative, gram-negative pathogens from various infection sources. Susceptibility was determined with CLSI broth microdilution and 2023 breakpoints. CR was defined as meropenem-nonsusceptible (NS). Only NME were analyzed because no CLSI breakpoint exists for IMI/REL against *Morganellaceae*. IMI-, IMI/REL-, and/or C/T-NS isolates were screened for β-lactamase genes.

**Results:**

Among 9524 collected NME isolates, 100 (1.0%) were CR. Among the most common species, CR rates ranged from 0.2% for *E. coli* to 2.1% for *K. pneumoniae* and *E. cloacae*. Of the 100 CR NME isolates, 90 were molecularly characterized: Two-thirds carried carbapenemases, almost all KPC; 13% carried only ESBL or AmpC, and in 20%, no acquired β-lactamases were detected (Figure 1). IMI/REL maintained activity against 76% of all CR NME, ≥50 percentage points higher than the comparator β-lactams and levofloxacin (Figure 2). IMI/REL inhibited 94% of 52 KPC-positive, 0 of 8 MBL- or OXA-48-like-positive, and 73% of 30 carbapenemase-negative CR NME isolates.

**Figure:**
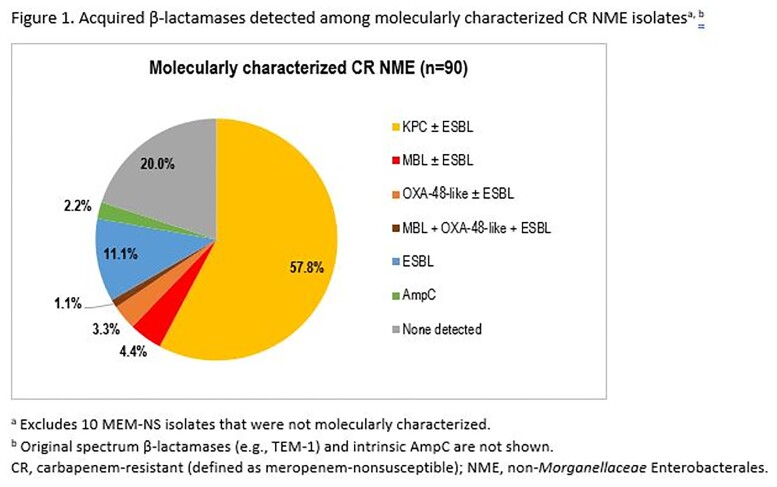

**Figure:**
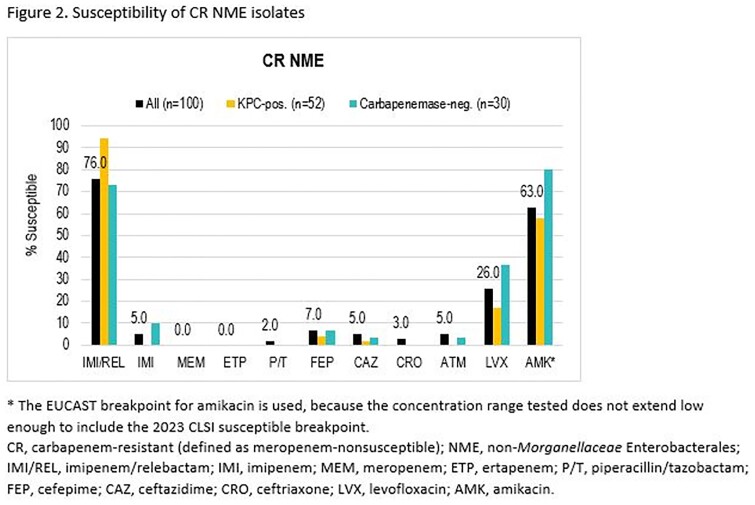

**Conclusion:**

Carbapenem resistance among clinical NME isolates collected in the US was largely due to carbapenemases, predominantly KPC while MBL or OXA-48-like were rare. IMI/REL represents an important therapy option for infections with CR NME that are KPC-positive or carbapenemase-negative.

**Disclosures:**

**Sibylle Lob, MD**, Merck & Co., Inc.: Honoraria **Mark G Wise, PhD**, Merck & Co., Inc.: Honoraria|Pfizer Inc.: Honoraria|Venatorx: Paid fees for conducting the study and abstract preparation **Fakhar Siddiqui, MD, MBA**, Merck & Co Inc.: Employee **Daniel F. Sahm, PhD**, Merck & Co., Inc.: Honoraria|Pfizer Inc.: Honoraria|Venatorx: Paid fees for conducting the study and abstract preparation

